# MetaPro-IQ: a universal metaproteomic approach to studying human and mouse gut microbiota

**DOI:** 10.1186/s40168-016-0176-z

**Published:** 2016-06-24

**Authors:** Xu Zhang, Zhibin Ning, Janice Mayne, Jasmine I. Moore, Jennifer Li, James Butcher, Shelley Ann Deeke, Rui Chen, Cheng-Kang Chiang, Ming Wen, David Mack, Alain Stintzi, Daniel Figeys

**Affiliations:** Department of Biochemistry, Ottawa Institute of Systems Biology, Microbiology and Immunology, Faculty of Medicine, University of Ottawa, Ottawa, ON Canada; Department of Paediatrics, CHEO Inflammatory Bowel Disease Centre and Research Institute, University of Ottawa, Ottawa, ON Canada

**Keywords:** Gene catalog, Gut microbiota, Metaproteomics, Metagenomics, Protein identification, Quantification

## Abstract

**Background:**

The gut microbiota has been shown to be closely associated with human health and disease. While next-generation sequencing can be readily used to profile the microbiota taxonomy and metabolic potential, metaproteomics is better suited for deciphering microbial biological activities. However, the application of gut metaproteomics has largely been limited due to the low efficiency of protein identification. Thus, a high-performance and easy-to-implement gut metaproteomic approach is required.

**Results:**

In this study, we developed a high-performance and universal workflow for gut metaproteome identification and quantification (named MetaPro-IQ) by using the close-to-complete human or mouse gut microbial gene catalog as database and an iterative database search strategy. An average of 38 and 33 % of the acquired tandem mass spectrometry (MS) spectra was confidently identified for the studied mouse stool and human mucosal-luminal interface samples, respectively. In total, we accurately quantified 30,749 protein groups for the mouse metaproteome and 19,011 protein groups for the human metaproteome. Moreover, the MetaPro-IQ approach enabled comparable identifications with the matched metagenome database search strategy that is widely used but needs prior metagenomic sequencing. The response of gut microbiota to high-fat diet in mice was then assessed, which showed distinct metaproteome patterns for high-fat-fed mice and identified 849 proteins as significant responders to high-fat feeding in comparison to low-fat feeding.

**Conclusions:**

We present MetaPro-IQ, a metaproteomic approach for highly efficient intestinal microbial protein identification and quantification, which functions as a universal workflow for metaproteomic studies, and will thus facilitate the application of metaproteomics for better understanding the functions of gut microbiota in health and disease.

**Electronic supplementary material:**

The online version of this article (doi:10.1186/s40168-016-0176-z) contains supplementary material, which is available to authorized users.

## Background

Intestinal microorganisms, namely the gut microbiota, have been shown to be important in multiple aspects of physiological processes relating to health and disease, including nutrition, metabolism, and immunity [1]. Host genetics and nutrition have been shown to affect the composition of gut microbiota; conversely, the microbial metabolites or the microbes themselves can also regulate host metabolic processes [1–4]. Disruption of the homeostasis surrounding these host-microbe interactions has recently been shown to participate in the development of many diseases including obesity, diabetes, and inflammatory bowel disease (IBD) [1]. Therefore, the gut microbiota is emerging as an important topic for public health and scientific researchers.

Next-generation sequencing (NGS) has been widely applied in gut microbiota studies using various experimental approaches [5]. Classically, NGS studies have focused on characterizing the taxonomic profile of the gut microbiota through targeted amplicon sequencing (e.g., 16S hypervariable regions) or through shotgun metagenomics. More recently, NGS has been used to characterize the metabolic potential of the gut microbiota through metatranscriptomic analysis [6]. While these approaches are quite useful, they are unable to demonstrate that the predicted biological processes are actually present in the gastrointestinal tract. Metaproteomics, which examines all the expressed proteins in a microbial community, has been shown to provide invaluable functional information for the gut microbiota [7]. Moreover, the application of proteomics and metaproteomics to host-gut microbe interactions will help to provide meaningful information on the roles of microbiota [8]. Although the first shotgun metaproteomic study of the gut microbiota was reported in 2009 [7], only few follow-up large-scale gut metaproteomic studies have been published. The reasons for this lack of progress have been discussed in several reviews [8–12] and include (1) the inability to detect low abundant proteins with current mass spectrometers coupled to the high diversity of the gut microbiota and, more importantly, (2) the low efficiency in identifying gut microbial peptide or proteins from acquired mass spectrometry (MS) spectra. The latter is mainly due to the lack of a suitable database (in terms of database coverage and size) for peptide-spectra matching (PSM) which is the key step for current bottom-up proteomic studies.

The ideal database for proteomics should be composed of all potentially expressed proteins in samples being analyzed with no additional spurious sequences; however, this is challenging for gut metaproteomics due to the enormous diversity and individual variations of gut microbiome [12]. The NCBI nr database is often used in metaproteomics due to its high sequence coverage. Unfortunately, its enormous size (~80 million entries) makes the database searching extremely time consuming and also provides less sensitive peptide identifications when using target-decoy approach for false discovery rate (FDR) filtering [13]. To overcome these weaknesses, customized databases are often employed and are composed of a list of known gut microbial genomes that are manually chosen and combined to generate a synthetic metagenome database [14–16]. However, it is unclear how representative these synthetic metagenome databases are as compared to the actual metagenome since many intestinal microbes are un-cultivable and/or their genomes have not been sequenced. In addition, the researcher’s choice of which bacteria taxa to include in the database is often arbitrary and may result in biases, which also makes the cross-study comparisons difficult. An alternative to manually curating a custom database is using a matched metagenome database. Briefly, either a “representative” subset or all the samples being investigated are subjected to metagenomic sequencing to compile a database of genes [14]. Since metagenomic sequencing introduces additional costs, it has not been generally performed in currently reported metaproteomic studies. These challenges have undoubtedly contributed to the fact that most metaproteomic publications only identify around 3000 proteins, which is far less than expected. Recently, Jagtap et al. [13] proposed a two-step database search strategy where a first search is performed against the target-only version of a database to generate a smaller refined database, which is then used for a second classical target-decoy database search [17]. This has been shown to increase the sensitivity of peptide identification and greatly increase the number of peptides and proteins identified. In a recent study by Tanca et al. [18] over 13,000 peptides corresponding to 9000 proteins were identified for mouse cecum samples by combining a matched metagenome database with the aforementioned two-step strategy. To the best of our knowledge, this is the highest protein identification for a single gut metaproteomic study to date. However, as mentioned above, the matched metagenome approach suffers from the need for metagenomic sequencing and the differences of databases used in different studies. Thus, a comprehensive gut microbial gene or protein database, which covers the expressed proteins for all gut microbial species, will make gut metaproteomics more affordable, comprehensive and comparable, which will largely promote wide application of metaproteomics in microbiome studies.

The Metagenomics of Human Intestinal Tract (MetaHIT) [19, 20] and the Human Microbiome Project (HMP) consortiums [21] have aimed to compile comprehensive gut microbial gene catalogs based on high-throughput sequencing to facilitate the analysis of multi-omic data. More recently, the close-to-complete human and mouse gut microbial gene catalog databases have been published and made freely available. Thus, in this study, we leveraged these gene catalogs as unified databases for protein identification in gut metaproteomics. We subsequently developed a high-performance and universal approach for gut *MetaPro*teome *I*dentification and *Q*uantification (MetaPro-IQ), which led to the quantification of approximately 120,000 peptides corresponding to >30,000 protein groups (defined as the cluster of proteins identified by the same set or a subset of peptides) in a single experiment. More importantly, since the gene catalog databases are centrally curated and publicly accessible, the current approach can be widely applied for gut microbiota studies to generate comparable results from different researchers. Thus, the MetaPro-IQ shows great potential as a universal approach which would largely promote the wide application of gut metaproteomics.

## Results and discussion

### Implementation of the MetaPro-IQ workflow for gut microbiota study

**Fig. 1:**
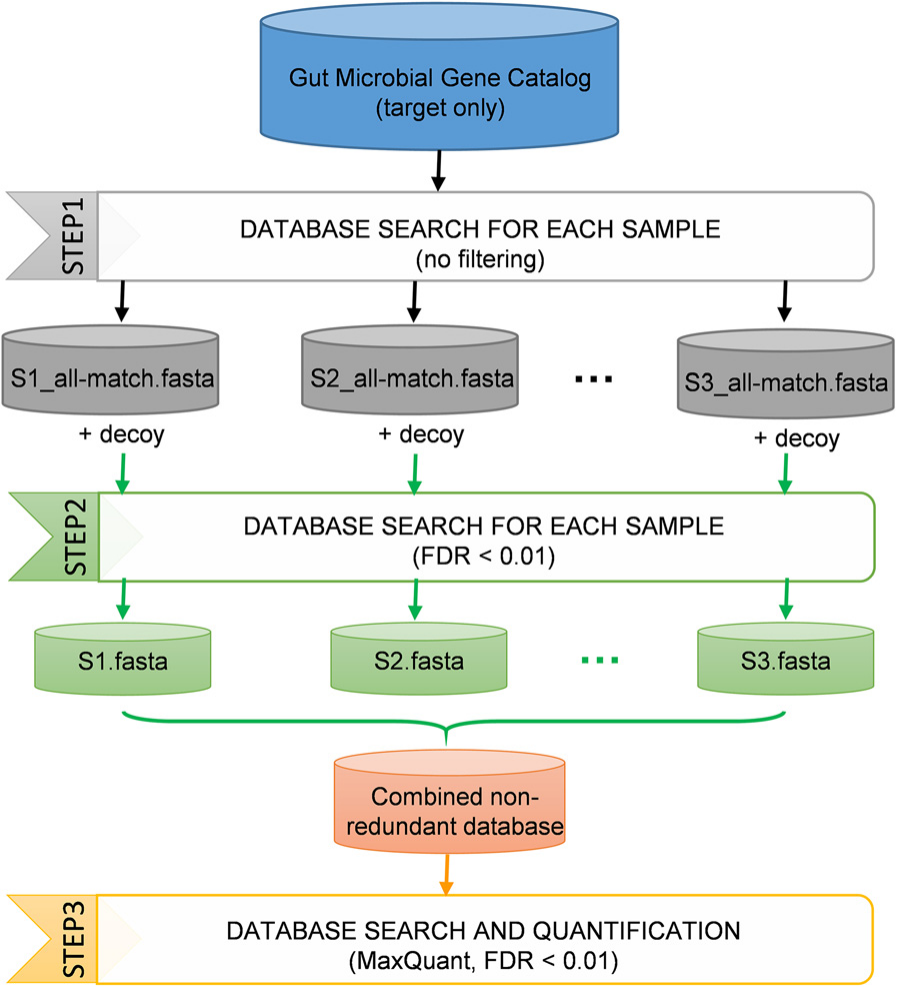
Overview of the MetaPro-IQ approach. The human or mouse gut microbial gene catalog is freely downloadable online [20, 24]. For the first two steps of the database search, each of the samples was processed individually. The identified protein sequences for all the samples were then combined to generate a refined small study-specific sub-database, which will be used for the final-step quantitative analysis using MaxQuant

In this study, we developed a high-performance metaproteomic workflow for enhanced gut microbial protein identification and quantification, namely MetaPro-IQ, using the close-to-complete human and mouse gut microbial gene catalog databases and iterative database search strategy. The implementation of MetaPro-IQ approach is illustrated in Fig. 1 and detailed as follows. Briefly, in the first step, a database search against the whole gene catalog database was performed to generate a “pseudo-metaproteome” database for each sample. A reduced database containing all possible proteins derived from peptide-spectrum matches was generated and hyphenated with reversed sequence for each sample and used for the second step, a typical target-decoy database search [17]. The confidently identified peptide and protein lists were then generated by applying strict filtering based on a FDR of 0.01, which is widely accepted for proteomic identifications. To obtain accurate and normalized quantitation information for all of the identified proteins across samples, the resulting protein lists for all samples were combined and de-duplicated to generate a “combined non-redundant database.” The latter was then imported into MaxQuant software for protein quantification using advanced MaxLFQ algorithms [22].

A gene/protein database is typically needed for the purpose of peptide identification and is readily available for most model organisms. A typical proteome database contains 5000–20,000 entries which leads to a reasonable database search time and FDR filtering sensitivity [17]. However, the gut microbiota consists of thousands of microbial species and most of them are remain largely unknown or lack any available genome or proteome sequences [23]. This makes the gut metaproteomic studies much more challenging. Therefore, in this study, we used the newly generated, well-annotated gut microbial gene catalog databases (available from http://meta.genomics.cn/ and http://gigadb.org/) [20, 24], to improve the database coverage for the gut microbial proteins. The human gut microbial gene catalog contains 9.9 million genes generated from >1200 metagenomic sequencing samples [20], and the mouse gut microbial gene catalog contains 2.6 million genes generated from 184 sequenced mouse samples of diverse genetic and environmental backgrounds [24]. These databases are the most comprehensive for human and mouse gut microbial genes to date, and more importantly, the application of these gene catalog databases allows the generation of unified protein lists from different studies, thus enabling easy cross-study comparisons. The latter is also one of the important disadvantages for the current gut metaproteomic studies as aforementioned.

The sizes of the abovementioned gene catalog databases are large (>10^6^ entries), which will greatly limit the database search sensitivity [13]. Iterative database search strategy has been previously shown to increase the sensitivity of peptide identification using huge databases, particularly for the metaproteomics and proteogenomics [13]. Thus, the MetaPro-IQ conducted a first-step database search against the whole gene catalog database, which generated a reduced database from the original gene catalog based on the acquired MS spectra in a sample. The reduced database was then used for a classical target-decoy database search to obtain confident peptide/protein identifications [13, 17].

In addition to the peptide/protein identification, quantification is another important aspect for proteomics. Spectra counting-based label-free quantification had been used for protein quantitation in many previous gut metaproteomic studies [7, 14, 15]. However, for data generated from high-resolution mass spectrometers, such as orbitraps, precursor-intensity-based algorithms have been shown to have superior accuracy, particularly when dynamic exclusion is enabled for spectra selection [22, 25]. MaxQuant is a widely used quantitative proteomics software implemented with the advanced MaxLFQ algorithm, which is based on precursor signal intensity and advanced delaying normalization across samples [22]. The MetaPro-IQ approach adopted MaxQuant for the final-step quantification analysis, which enabled accurate quantifications of the identified microbial proteins.

**Fig. 2:**
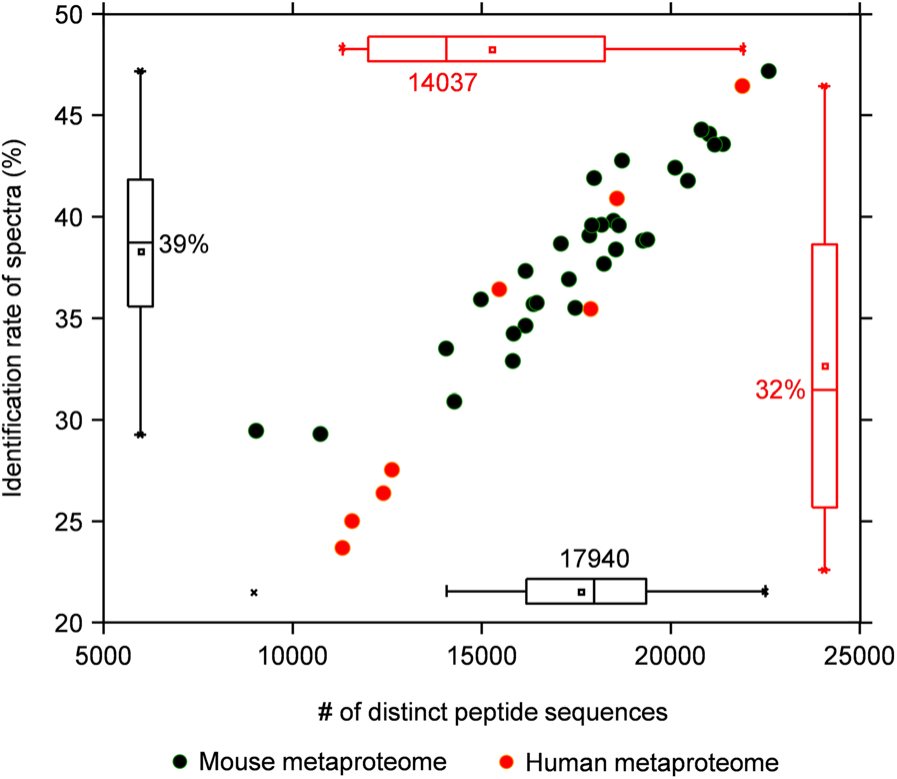
Peptide identification of mouse stool and human MLI metaproteome datasets. Scatter plots and box plots showing the number of identified distinct peptide sequences (*x-axis*) and identification rate (*y-axis*) for each sample in mouse stools (*black*) or human mucosal-luminal interface (*red*) samples. The identification rate was calculated by dividing the identified MS/MS by total acquired MS/MS. The median (*central thick lines*), 25 and 75 % quartile ranges (*box width*), and upper and lower limits (*error bar*) were shown in the box plot. The median values were indicated besides each of the box plot

We then applied the MetaPro-IQ approach on two distinct datasets. The first dataset was from a mouse metaproteomic study wherein a total of 32 stool samples were collected from either high-fat diet (HFD)- or low-fat diet (LFD)-fed mice. The bacteria from stool samples were then processed, trypsin digested, and subjected to a 4-h gradient MS run for each sample on the Q Exactive mass spectrometer. A total of 121,588 distinct peptide sequences and 30,749 protein groups were quantified with a median of 17,940 peptide identifications for each sample (39 % of the total acquired tandem MS spectra, Fig. 2). The second dataset was generated from human mucosal-luminal interface (MLI) samples. Briefly, the MLI samples were collected during endoscopy, from the ascending colon of eight different children. The bacteria were isolated, processed, and subjected to the same MS analysis as described for the mouse metaproteome study. We quantified 67,186 distinct peptides corresponding to 19,011 protein groups, with a median identification rate of 32 % (23–46 %) and 15,210 peptide sequences (11,310–21,889) for each sample (Fig. 2 and Additional file 1: Table S2). To our best knowledge, the results we obtained represent the largest number of gut microbial peptide and protein identifications from a single experiment. In addition, the MS identification rate in our study is comparable to those achieved in mono-culture microbial proteomic studies [15, 26].

### MetaPro-IQ reached comparable performance with matched metagenome strategy

**Fig. 3:**
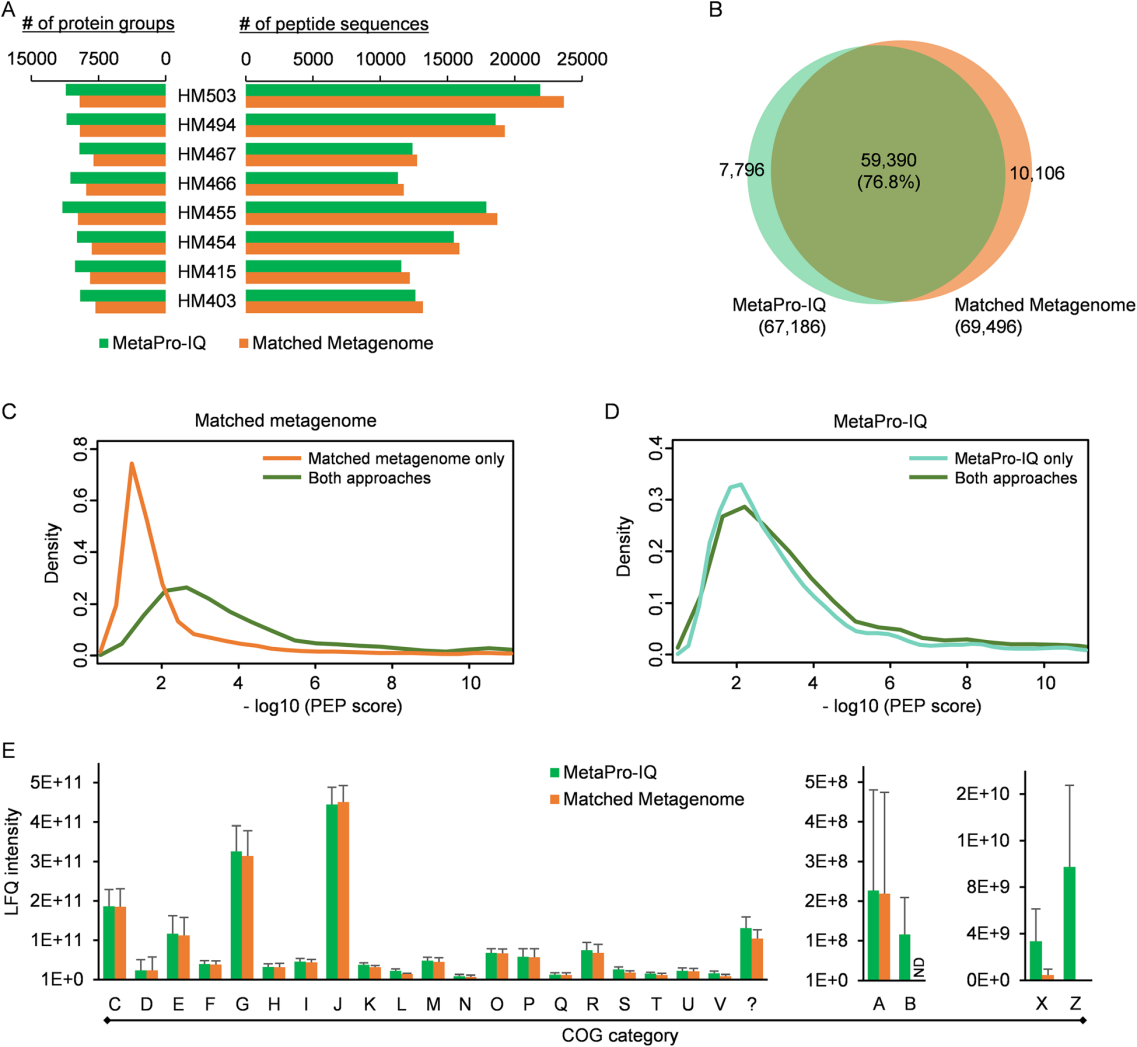
Comparison between MetaPro-IQ and matched metagenome approaches. **a** Peptide and protein group identification for each of the human MLI samples. **b** Venn diagram showing the overlap of identified peptides between the MetaPro-IQ and matched metagenome approaches for the whole dataset. The overlap percentage in the bracket was calculated by dividing the overlapped peptides with the total identified peptides by either MetaPro-IQ or matched metagenome approaches. **c** Posterior error probability (PEP) score distribution of peptides only identified with the matched metagenome approach. **d** PEP score distribution of peptides only identified with MetaPro-IQ approach. **e** Distribution of COG categories. LFQ intensity was used for the analysis, and mean ± SD was plotted. Each *letter* shows one COG category according to the standard naming in NCBI website and also shown in Additional file 1: Table S7. *ND* not detected. *Question mark* (?) denotes proteins without a COG assignment

Matched metagenome database has been shown to be a good choice for metaproteomic study with reasonable database size and coverage [14, 18], although this approach suffers from the need for metagenomic sequencing. The current workflow aimed to skip the need for metagenomic sequencing and instead generate a database akin to a pseudo-expressed metagenome with the current MS sampling sensitivity. To evaluate whether similar performance could be reached to the matched metagenome strategy, we conducted metagenomic sequencing for all the eight human MLI samples. A total of 243 million high-quality paired-end 100-bp Illumina sequencing reads were generated (25–35 million paired-end reads for each sample, Additional file 1: Table S3). The genes were then predicted using the previously established MOCAT pipeline [27], which generated an average of 119,777 non-redundant genes per sample (Additional file 1: Table S3). The resulting genes for each sample were then used for matched metagenome database searches for peptide/protein identification. The matched metagenome strategy quantified 69,496 peptide sequences corresponding to 16,415 protein groups for the whole dataset (Additional file 1: Table S2). As mentioned above, the MetaPro-IQ approach using the human gut microbial gene catalog database quantified 67,186 peptide sequences corresponding to 19,011 protein groups. The average identification rate of the acquired MS spectra was 34 and 33 % for the matched metagenome and the MetaPro-IQ approaches, respectively. The MetaPro-IQ approach identified comparable number of peptides but more protein groups for each of the samples (Fig. 3a). More than 76 % of the total identified peptide sequences were identified by both approaches, with 13 % (10,106 peptides) of all the peptides only identified by the matched metagenome approach and 10 % (7796 peptides) only by MetaPro-IQ approach (Fig. 3b). Among the peptides only identified with the matched metagenome approach, 75 % (7554 peptides) were present in the whole gene catalog database. However, further examination of these peptides revealed an obvious lower quality of identification (represented as posterior error probability (PEP) scores) as compared to those identified with both approaches (Fig. 3c). Among the peptides only identified with MetaPro-IQ, 27 % (2106 peptides) of the peptides were present in the matched metagenome databases. In contrast with the peptides only identified using matched metagenome approach, there was no obvious difference in PEP distribution for those only identified using MetaPro-IQ as compared to those identified by both approaches (Fig. 3d). These findings suggest that the MetaPro-IQ workflow using the gene catalog database performed better for peptide and protein identification than the workflow using the matched metagenome database.

To compare the abilities of the two approaches for extracting functional information, all the quantified proteins were annotated with Clusters of Orthologous Group (COG) categories. Twenty-three COG categories were observed with the matched metagenome approach, which were all found with the MetaPro-IQ approach. There is no obvious difference in the relative abundance of the high abundant COG categories between the two approaches (Fig. 3e). Several COG categories such as B, Z, and X were solely present or with obviously higher observed LFQ intensity using the MetaPro-IQ approach (Fig. 3e). This may result from the lack of low abundant genes in matched metagenome databases due to inadequate sequencing depth. The low abundant genes may have relatively high protein-expression levels which are detectable using mass spectrometers, and thereby were identified by MetaPro-IQ approach.

To examine whether the above observations are dataset dependent, the murine fecal metaproteome dataset (MFM; two replicates with two runs for each replicate) from the study of Tanca et al. [18] were re-analyzed with the MetaPro-IQ workflow. In total, we quantified 19,497 peptides and 4549 protein groups for replicate 1 and 19,972 peptides and 4630 protein groups for replicate 2. More than 92 % of the peptides were quantified for both replicates (Additional file 2: Figure S1A). Tanca et al.’s study, using a matched metagenome database search strategy, identified 14,085 peptides for replicate 1 and 15,669 peptides for replicate 2 with an overlap of 63 % [18]. Compared to the matched metagenome strategy, the MetaPro-IQ workflow identified more peptides with a better overlap between replicates for their dataset. In addition, a Pearson’s correlation coefficient of 0.89 was obtained between the two replicates, and more than 0.86 between runs (two mass spectrometry runs were conducted for each replicate in Tanca et al.’s study, Additional file 2: Figure S1B–F), which is also in agreement with the findings in their study.

Taken together, the MetaPro-IQ metaproteomic workflow using the gut microbial gene catalog database showed better performance for identifying gut microbial proteins, when compared to the workflow using a matched metagenome database. MetaPro-IQ allows high efficient protein identification from MS spectra in metaproteomics without the need for prior metagenomic sequencing (greatly reduces the experimental cost) and is readily applicable for all researchers from various disciplines.

### MetaPro-IQ approach revealed metaproteome response of gut microbiota to diet in mice

**Fig. 4:**
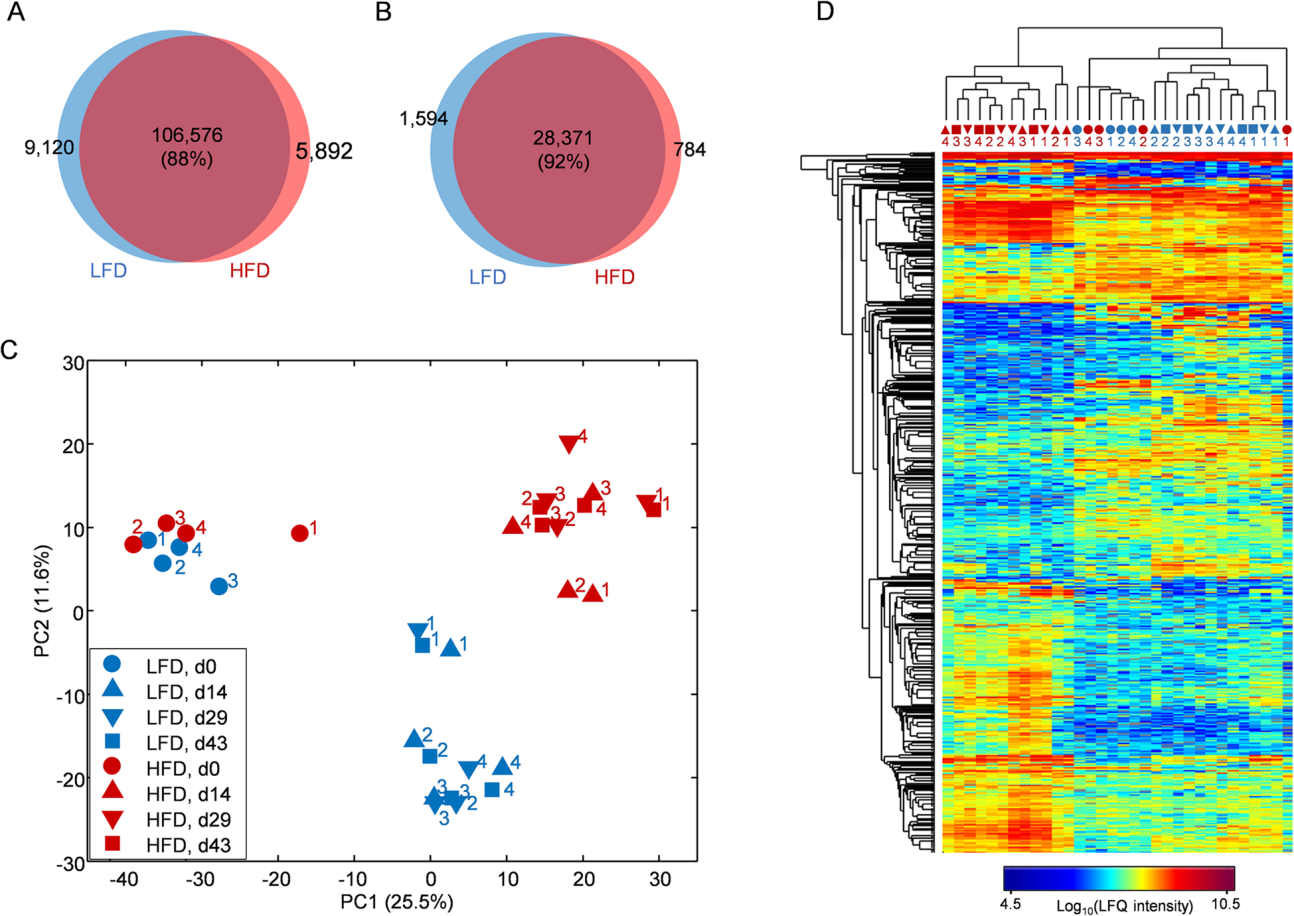
Response of mouse gut metaproteome to high-fat diet feeding. Venn diagrams showing the overlap of identified peptides (**a**) and protein groups (**b**) between LFD and HFD groups. The overlap percentage in the *bracket* was calculated by dividing the overlapped number by the total identified ones for the whole dataset. **c** PCA score plot. Missing values were imputed with nearest-neighbor method in MATLAB. *Numbers in the graph* indicate the individual mouse ID in each group. **d** Heat map of the identified 849 key proteins responding to high-fat feeding. The *color of the spot* corresponds to the log_10_-transformed LFQ intensity of each protein. Both column and row clustering were generated based on Euclidean distance using the average linkage method. The sample codes (*column*) follow the same scheme as in panel (**c**)

The alteration of gut microbiota in HFD-fed animals has been considered to be involved in the development of HFD-induced metabolic disorders [28]; however, the mechanism remains unclear. In-depth metaproteomic analysis of the functional changes in the microbiota during HFD feeding may provide valuable information on diet-microbiota-host interactions. Thus, in this example, the response of the gut microbiota to diet in mice was studied using the MetaPro-IQ metaproteomic approach. Briefly, eight mice were fed with either HFD or LFD for 43 days. As expected, the HFD-fed mice gained significantly more body in 4 weeks (Additional file 2: Figure S2). Stool samples were collected at days 0, 14, 29, and 43 of the trial and subjected to metaproteomic analysis. In total, we quantified 30,749 protein groups from 32 samples, and a large overlap between the HFD and LFD groups for both quantified peptides (88 %) and protein groups (92 %) was observed (Fig. 4a, b). Relatively fewer unique peptides and proteins were quantified in HFD groups compared to the LFD groups, which might be due to the reduced microbial diversity in HFD-fed mice [29]. The principal component analysis (PCA) score plot showed three obvious clusters corresponding to the baseline, HFD-fed mice, and LFD-fed mice (Fig. 4c). The HFD and LFD diets are matched in terms of ingredients with different proportions of fat (Additional file 1: Table S4) and are different from the normal chow diet used at the baseline. Since the samples from days 14, 29, and 43 clustered closely together under both HFD and LFD feeding conditions (Fig. 4c), the data for each group at days 14, 29, and 43 were combined and compared to identify key proteins relevant to dietary fat composition, using a two-sample *t* test with a Benjamini-Hochberg FDR correction. A total of 849 significantly changed proteins (*q* < 0.05) were identified with 438 increased and 411 decreased, respectively, in the HFD group compared to LFD group (Fig. 4d and Additional file 1: Table S5). Among these 849 significantly changed proteins, 583 proteins were found to be significantly different between baseline and HFD-fed mice and 246 between baseline and LFD-fed mice (Additional file 1: Table S5). Hierarchical clustering analysis with those key proteins showed that the samples from the same mouse clustered together in the LFD group, while this was not the case in the HFD group (Fig. 4d). In addition, the metaproteome patterns of LFD-fed mice clustered with the normal-chow-diet-fed mice (baseline), far apart from the HFD groups, which also suggest that the identified significant proteins might be related to the dietary fat composition. COG analysis showed that the most abundant COG categories were G (carbohydrate transport and metabolism), C (energy production and conversion), and J (translation, ribosomal structure, and biogenesis) in both HFD- and LFD-fed mouse microbiota (Additional file 2: Figure S3), which is in agreement with previous studies [15]. Among the 23 COG categories, category O (posttranslational modification, protein turnover, chaperones) was significantly increased in HFD group, while category S (function unknown) was significantly decreased (Additional file 2: Figure S3). Protein posttranslational modifications have been considered to present under various pathological conditions or following exposure to toxic agents in cells [30]. Our findings suggest that the microbiota in the gut of the HFD-fed mice may experience substantial stress from either the host or the diet, which may be an important part of the disrupted homeostasis of diet-microbiota-host interactions during high-fat feeding.

More than 94 % of the key microbial proteins have a greater than twofold change between HFD and LFD groups, while thirty eight of them have more than a 100-fold change, representing the major responders to HFD (Additional file 1: Table S5). Proteins S-Fe9_GL0178463 and S-Fe7_GL0107273 were the most markedly changed proteins with more than 1000-fold decrease in the HFD group, and both of them were annotated as hypothetical proteins from *Eubacterium plexicaudatum* (Additional file 1: Table S6). Further examination of peptide identifications revealed that protein S-Fe9_GL0178463 had high-quality PSMs (scores up to 323) in all samples in the LFD group, but no PSM was obtained for HFD groups (Additional file 2: Figure S4). The MaxQuant software has been designed to identify and quantify peptides from precursor ions without any MS/MS scan, by matching to the high-quality PSMs in paralleled samples based on retention time and mass-to-charge (*m*/*z*) ratio [22]. This resulted in an average of three peptides quantified for the samples in HFD group (Additional file 2: Figure S4). The lack of PSM for protein S-Fe9_GL0178463 in the HFD group might be the result of low protein levels in the samples, such that their precursor ions were not abundant enough to be selected by mass spectrometer for fragmentation. However, the precursor ions were still present allowing accurate quantification and thereby selection of key proteins with huge differences between the different groups. This scenario, where certain peptides/proteins are almost totally absent from a single group, is expected to be common in gut microbial studies, where some species may be substantially inhibited by other species or environmental compounds in one group as compared to another.

**Fig. 5:**
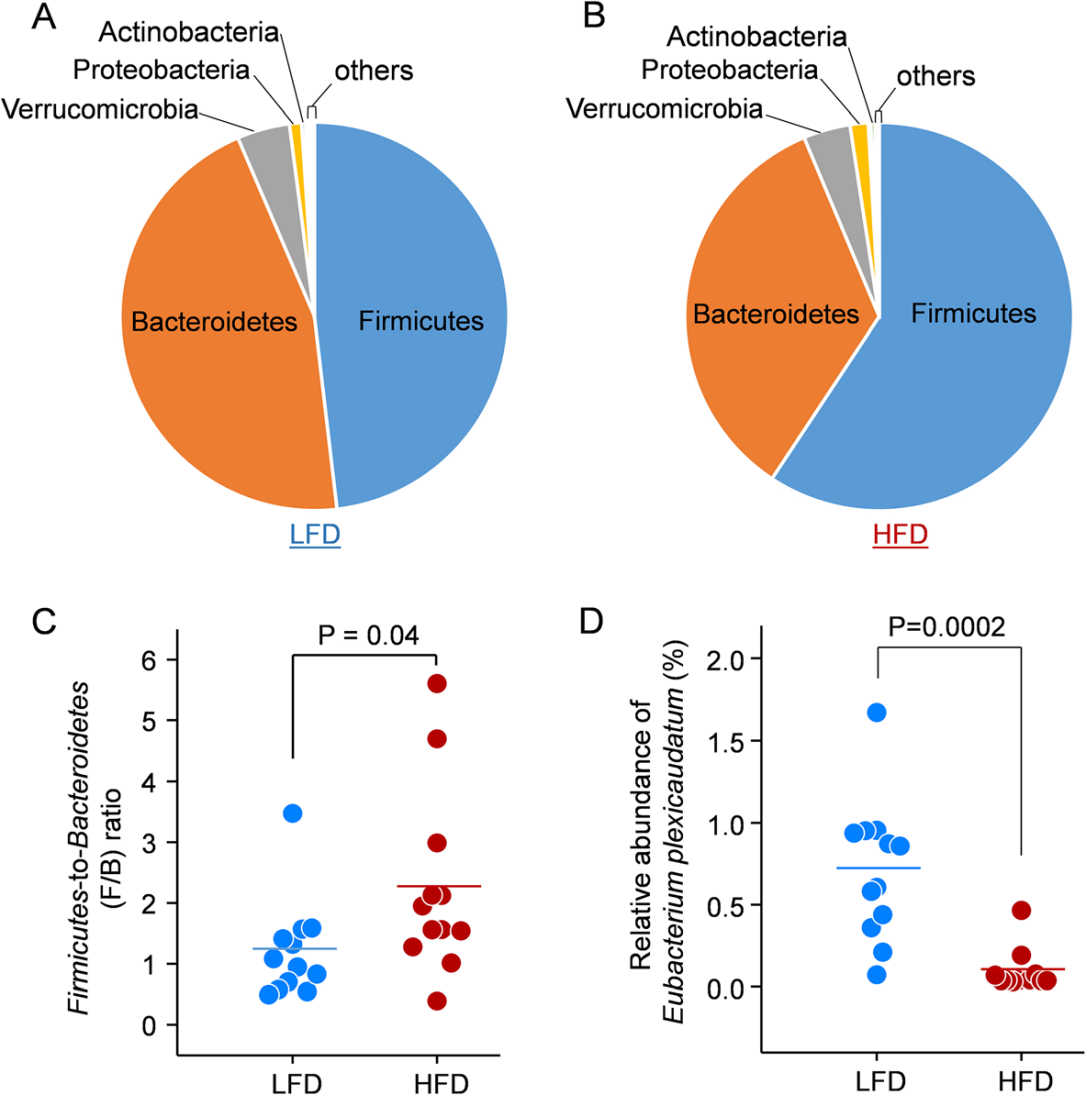
Taxonomy analysis of the effects of HFD feeding on mouse gut metaproteome. Phylum level distributions of LFD group (**a**) and HFD group (**b**) were shown. The total intensities of all identified unique peptides for each phylum in all samples in each group were summed for pie chart plotting. **c** *Firmicutes*-to-*Bacteroidetes* ratios in LFD- and HFD-fed mouse gut metaproteomes. **d** The relative abundance of *Eubacterium plexicaudatum* based on the unique peptides identified. The total intensities of all identified unique peptides were summed and normalized with total intensity in each sample. Significance was examined using a Mann-Whitney test, and *p* values were indicated

Taxonomy analysis was performed using the unique peptide-based approach as described previously [31, 32]. In agreement with previous metaproteomic and metagenomics studies [15, 18, 33], *Firmicutes*, *Bacteroidetes*, *Verrucomicrobia*, *Proteobacteria*, and *Actinobacteria* were the most abundant phyla in mouse stool (Fig. 5a, b). The *Firmicutes*-to-*Bacteroidetes* (F/B) ratio was significantly increased in HFD-fed mice (Fig. 5c), which was also in agreement with previous metagenomic studies [34]. A total of 595 unique peptides were found for *E. plexicaudatum*, which was identified to have two proteins with a >1000-fold decrease in the HFD group. The relative abundance of this species, which was represented as the sum intensity of all the unique peptides, was also significantly decreased in the HFD group by sevenfold compared to the LFD group (Fig. 5d). *E. plexicaudatum* is a butyrate-producing bacterium [35], which has been shown to protect the integrity of the intestinal epithelium and exert anti-inflammatory effects [28, 36, 37]. Butyrate-producing bacteria are reported to be decreased in HFD-fed animals and in some human diseases such as obesity and IBD [28, 36, 38, 39]. The current study suggests that the proteins S-Fe9_GL0178463 and S-Fe7_GL0107273 may participate in the response to HFD in *Eubacterium*, which is worthy of further investigation.

## Conclusions

MetaPro-IQ approach provides a universal, easy, and high-performance metaproteomic workflow for studying human or mouse gut microbiota and dramatically improves microbial protein identification and quantification. The workflow was established based on the usage of the close-to-complete gut microbial gene catalog database and an iterative database search. These enabled high protein-database coverage and better sensitivity for peptide identification. More importantly, it allows easy comparison for the results obtained from different studies. The MetaPro-IQ approach also makes matched metagenome sequencing unnecessary for general gut metaproteomic studies. Moreover, the workflow generated quantitative results based on precursor intensity and advanced normalization method, which allows accurate comparison across samples.

The limitation of the MetaPro-IQ also happens to the gene catalog databases since they are mainly generated from stool samples and will not represent all types of intestinal microbiomes, for example, the biopsy microbiomes. For uncommon samples, the gene catalog database could be enhanced with additional matched sample metagenomic sequencings, and thus will improve the performance of MetaPro-IQ. In addition, the current gut microbial gene catalog databases represent well for the gut-related bacteria and archaea; the studies focusing on other taxa such as fungi and virus will need further addition of their protein sequences or alternative databases. However, the concept of MetaPro-IQ is applicable for any research as long as the gene catalog or reference protein database is available.

## Methods

### Mouse stool sample collection and processing

The animal experiments were performed at the Ottawa Hospital Research Institute and conducted in strict accordance with the guidelines on the Care and Use of Experimental Animals of Canadian Council on Animal Care (CCAC). The animal use protocol (2009-012) was approved by the Animal Care Committee at the University of Ottawa. A total of eight male C57BL/6J mice (Charles River, Sherbrooke, QC) were housed individually in the same room at 25 °C with a strict 12-h light/dark cycle. Food and water were available ad libitum. Mice were acclimatized to the facility for 2 weeks and fed a normal chow diet (containing 18 % fat by energy; Harlan Laboratories, Inc., Madison, WI) and then randomly divided into two groups (*n* = 4/group). One group of mice was fed a high-fat diet (containing 39.7 % fat by energy; TestDiet, St. Louis, MO), and the other group a low-fat diet (containing 15.8 % fat by energy; TestDiet) for 43 days. Body weight for each mouse was monitored weekly. Stool samples were collected at days 0, 14, 29, and 43 and stored at −80 °C until analysis.

For proteomic analysis, bacterial proteins were extracted from the stool samples. Briefly, stool samples (~1 g) were suspended in 1.5 mL ice-cold PBS (pH 7.0) with thorough vortexing. The slurries were centrifuged at 300*g*, 4 °C for 5 min. Supernatants were carefully collected, and the pellets were subjected to the above procedure three times. All the supernatants for each sample were then combined followed by three more centrifugations at 300*g*, 4 °C for 5 min to remove debris. The supernatant was then centrifuged at 14,000*g*, 4 °C for 20 min to pellet bacterial cells. The pellet was then re-suspended in fresh PBS and washed another three times. The bacterial cells were then lysed with 4 % sodium dodecyl sulfate (SDS) and 6 M urea in 50 mM Tris-HCl buffer (pH 8.0) with sonication. SDS was removed by protein precipitation in acidified acetone/ethanol buffer at −20 °C overnight. The precipitated proteins were dissolved in 6 M urea in 50 mM ammonium bicarbonate (pH 8) for trypsin digestion.

### Human MLI sample collection and processing

Eligible subjects were under 18 years of age and scheduled to undergo diagnostic colonoscopy (Additional file 1: Table S1). The protocol was approved by the Research Ethics Board of the Children’s Hospital of Eastern Ontario (CHEO). Colonoscopy preparation was done as per standard protocol modified to 1 day [40]. During colonoscopy, once the proximal ascending colon was intubated, any fluid and loose debris was aspirated and discarded. Thereafter, sterile water was flushed onto the mucosa to dislodge adherent mucus from mucosal epithelial cells and the mixture was then aspirated into a sterile container through the colonoscope. The samples were immediately placed on ice and transported to the lab for processing.

To remove any debris in the aspirate sample, a first centrifugation at 700*g*, 4 °C for 5 min was performed and the supernatant was transferred into a new tube. The bacterial cells were then collected with a centrifugation at 14,000*g*, 4 °C for 20 min. The pellets were then used for protein extraction according to the procedures described above.

### Liquid chromatography-tandem mass spectrometry

In-solution trypsin digestion for bacterial proteins recovered from both mouse stool and human MLI samples was conducted as described previously [41]. Briefly, the proteins were first reduced and alkylated with 10 mM dithiothreitol (DTT) and 20 mM iodoacetamide (IAA), respectively. The urea concentration was then diluted to <1 M with 50 mM ammonium bicarbonate. Trypsin (Worthington Biochemical Corp., Lakewood, NJ) was then added at a protein-trypsin ratio of 50: 1 (*w*/*w*) for digestion overnight with agitation at 37 °C. The tryptic digest was desalted with a 10-μm C18 column and eluted with 80 % acetonitrile/0.1 % formic acid. The eluent was then evaporated with a Speed-Vac concentrator and tryptic peptides dissolved in 0.1 % formic acid for mass spectrometry analysis.

Tryptic peptides equivalent to 4 μg of proteins were loaded for liquid chromatography-tandem mass spectrometry (LC-MS/MS) analysis on a Q Exactive mass spectrometer (ThermoFisher Scientific Inc.). The separation of peptides was performed on an analytical column (75 μm × 50 cm) packed with reverse phase beads (1.9 μm; 120-Å pore size; Dr. Maisch GmbH, Ammerbuch, Germany). A 4-h gradient was performed from 5 to 35 % acetonitrile containing 0.1 % formic acid at a flow rate of 200 nL/min. The instrument method consisted of one full MS scan from 300 to 1800 *m*/*z* followed by data-dependent MS/MS scan of the 12 most intense ions, a dynamic exclusion repeat count of 2, and repeat exclusion duration of 30 s. All data were recorded with the Xcalibur software and exported as.raw format for further analysis.

### Metagenomic DNA extraction, sequencing, and gene prediction

Total DNA was extracted from intestinal aspirate samples using the Fast DNA spin kit (MP Biomedicals, Santa Ana, CA) and using a FastPrep-24 (MP Biomedicals). Briefly, MLI samples were thawed and contents pelleted by centrifuging at 14,000*g* for 10 min in a bench top centrifuge. The pellets were re-suspended in 1 mL of cell lysing solution (CLS)-TC and subjected to two mechanical lysis cycles at speed 6.0 for 40 s. The extracted DNA was then used to construct sequencing libraries using an Illumina TruSeq DNA Sample Prep kit v3 according to the manufacturer’s instructions. The sequencing was performed on an Illumina HiSeq 2000 (generating paired-end 100-bp reads) at the Génome Québec Innovation Centre, McGill University (Montreal, Canada).

Gene prediction was performed on each sample individually using the previously published MOCAT pipeline [27]. Briefly, raw reads were first filtered and trimmed to remove sequencing adapters and low quality reads. Reads with human origin were removed using SOAPAligner 2 against the human genome database (hg19) [42]. The remaining reads were then used for scaftig assembly and assembly revision to generate assembled sequences for gene prediction with the MetaGeneMark algorithm [43]. The resulting gene sequence lists for each of the sample were then compiled into FASTA files and used as a matched metagenome database for benchmarking MetaPro-IQ.

### Bioinformatics for metaproteome data analysis

#### Implementation of MetaPro-IQ approach

The implementation of the MetaPro-IQ approach is illustrated in Fig. 1 and details are highlighted in the “Results and discussion” section. The human and mouse gut microbial gene catalog databases were downloaded from the IGC website (http://meta.genomics.cn/) and the GigaScience Database (http://gigadb.org/dataset/view/id/100114/token/mZlMYJIF04LshpgP), respectively [20, 24].

In MetaPro-IQ, the first- and second-step database searches were carried out with X! Tandem (release 2015.04.01) [44, 45], and the third step was carried out with MaxQuant software (version 1.5.2.8) [46]. For the X! Tandem database search, each raw file obtained in the current study or from Tanca et al.’s study (downloaded from PeptideAtlas Repository at http://www.peptideatlas.org/PASS/PASS00355) was converted into mgf format with an in-house software platform. The tandem search was performed with up to two miss-cleavages (trypsin/P), carbamidomethylation of cysteine as a fixed modification, and oxidation of methionine as a potential modification. A fragment ion tolerance of 20 ppm and a parent ion tolerance of 10 ppm were used. All matched protein sequences for the first-step search were extracted as the sample-specific database (sample_all-macth.fasta). The X! Tandem outputs of the target-decoy database search (step 2) were summarized with an in-house software to generate an identified protein list at a FDR cutoff of 0.01 and maximum expect value of 0.05. The resulting protein list for all samples was then combined, and duplicates were removed for generating a “combined non-redundant database” to use for protein quantification using MaxQuant. Similar peptide identification parameters with X! Tandem database searches were used for MaxQuant (parameter files were uploaded together with the raw data and result files to ProteomeXchange). For quantification, the LFQ algorithm was used for label-free quantification. Both razor and unique peptides were used for protein quantification, and the minimum ratio count was set as 1. An alignment retention time window of 20 min and match time window of 5 min were applied to match the same accurate masses between different runs. Proteins identified by the same set or a subset of peptides were grouped together as one protein group.

#### Matched metagenome database search strategy

Matched metagenome database searches were performed with exactly the same parameters as the MetaPro-IQ approach, except that the metagenome database (gene sequences) was used for each sample instead of the whole gene catalog database. To ensure a fair comparison, the same multi-step database search strategy and MaxQuant quantification was performed. The outputs of MaxQuant (namely the results included in the txt folder) were then compared between the matched metagenome and MetaPro-IQ approaches.

### Functional annotation into COGs

All the quantified protein sequences were aligned against the COG database (ftp://ftp.ncbi.nih.gov/pub/COG/COG2014/data) with DIAMOND using default parameters (e-value cutoff of 0.001) [47, 48]. The best hit for each query was selected for annotation, and the COG id, name, and category information for each of the matched sequences were extracted from the annotation file of the COG database. If the leading protein (defined as the top rank protein in a group; ranking is based on the number of peptide sequences, the number of PSMs, and the sequence coverage) in a protein group had no match for COG id, the others were checked for the existence of any match. The LFQ intensity of all the protein groups annotated with the same COG category were summed together to represent the COG category abundance for each sample.

### Taxonomic analysis

Taxonomic analysis was performed based on taxon-specific unique peptides using Unipept (version 3.1) [31]. All of the quantified peptide sequences from MaxQuant were imported into the Unipept for analysis with the “Equal I and L” and “Advanced miss cleavage handling” options allowed. The results were then exported as a table format. The relative abundance of each taxon (namely phylum or species in this study) is represented as the sum of intensity for all the unique peptides found with Unipept.

### Multivariate statistical analysis

The protein group results from MaxQuant were first imported into Perseus (version 1.5.2.4) to remove any contaminants, reverse sequences, and those identified only by site. The LFQ intensity was log_10_-transformed and used for statistical analysis. Only those protein groups with valid LFQ intensity values in at least 29 of the 32 samples (>90 %) were kept for subsequent statistical and multivariate analysis. This resulted in a stringently quantified dataset of 5299 protein groups for the bacteria from 32 stool samples. Hierarchical clustering analysis was also conducted in Perseus with default parameters. Significant proteins between HFD and LFD groups were identified by a two-sample *t* test with Benjamini-Hochberg FDR correction. Those proteins with a FDR corrected *p* value (*q* value) of <0.05 were considered as significant proteins.

PCA was performed in MATLAB (version 2010b, The MathWorks Inc.), and the first two PCs were used for generating the score plot. A prior missing value imputation was done with the nearest-neighbor method in MATLAB with the *knnimpute* function (http://www.mathworks.com/help/bioinfo/ref/knnimpute.html) [49].

## Abbreviations

COG, Clusters of Orthologous Groups; DTT, dithiothreitol; FDR, false discovery rate; HFD, high-fat diet; HMP, Human Microbiome Project; IAA, iodoacetamide; IBD, inflammatory bowel disease; LC-MS/MS, liquid chromatography-tandem mass spectrometry; LFD, low-fat diet; MetaHIT, Metagenomics of Human Intestinal Tract; MetaPro-IQ, metaproteome identification and quantification; MLI, mucosal-luminal interface; PCA, principal component analysis; PEP, posterior error probability; PSM, peptide-spectra matching; SDS, sodium dodecyl sulfate

## Additional files

Additional file 1: Tables S1–S7.
**Table S1.** Clinical information of the eligible pediatric volunteers involved in the mucosal-luminal interface sample collection. **Table S2.** Comparison of peptide and protein identifications between the MetaPro-IQ and matched metagenome approaches. **Table S3.** Summary of metagenomic sequencing and gene prediction for pediatric mucosal-luminal interface samples. **Table S4.** Ingredient comparison of high-fat and low-fat diets. **Table S5.** List of 849 significant proteins responding to the high-fat feeding. **Table S6.** Best hits of BLASTP alignments against NCBI nr database. **Table S7.** Names and one-letter codes for identified COG categories. (XLSX 611 kb) Additional file 2: Figure S1–S4.
**Figure S1.** Analysis of murine fecal metaproteome data in the study of Tanca et al. using MetaPro-IQ approach. (A) Venn diagrams depicting the overlap of identified peptides between the two replicates; (B) scatter plot illustrating the Pearson’s correlation between the LFQ intensity of all peptides identified in both replicates; (C) and (E) showing the overlap of identified peptides between two runs for replicate 1 and replicate 2, respectively; (D) and (F) showing the Pearson’s correlation between the LFQ intensity of all peptides identified in both runs for replicate 1 and replicate 2, respectively. Detailed information on the sample information of murine fecal metaproteome (MFM) data was described in the study of Tanca et al. **Figure S2.** Effects of diet on the mouse body weight gain. Statistical differences were examined with two-sample *t* test. Mean ± SD was shown. **p* < 0.05. **Figure S3.** COG category distributions of mouse stool microbial proteins. The COG category distributions of all proteins were shown. LFQ intensity was used for the analysis, and mean ± SEM was plotted. Each letter shows one COG category according to the standard naming in NCBI website and also shown in Additional file 1: Table S7. Question mark (?) denotes proteins without a COG assignment. Statistical analysis was performed using a two-sample *t* test with a Benjamini-Hochberg FDR correction. ***FDR-corrected *P* < 0.001. **Figure S4.** MS/MS count and peptide distribution of protein S-Fe9_GL0178463. The number of identified MS/MS and peptides for each sample was shown in the bar chart. The yellow box highlighted the samples with peptide identification only by matching instead of by MS/MS. (DOCX 583 kb)
