# Supplementary material for: MetaPro-IQ: a universal metaproteomic approach to studying human and mouse gut microbiota
Source: Microbiome. 2016 Jun 24;4:31. doi: 10.1186/s40168-016-0176-z (PMC4919841; doi:10.1186/s40168-016-0176-z)

**Supplementary Figures**

Supplementary Figure S1
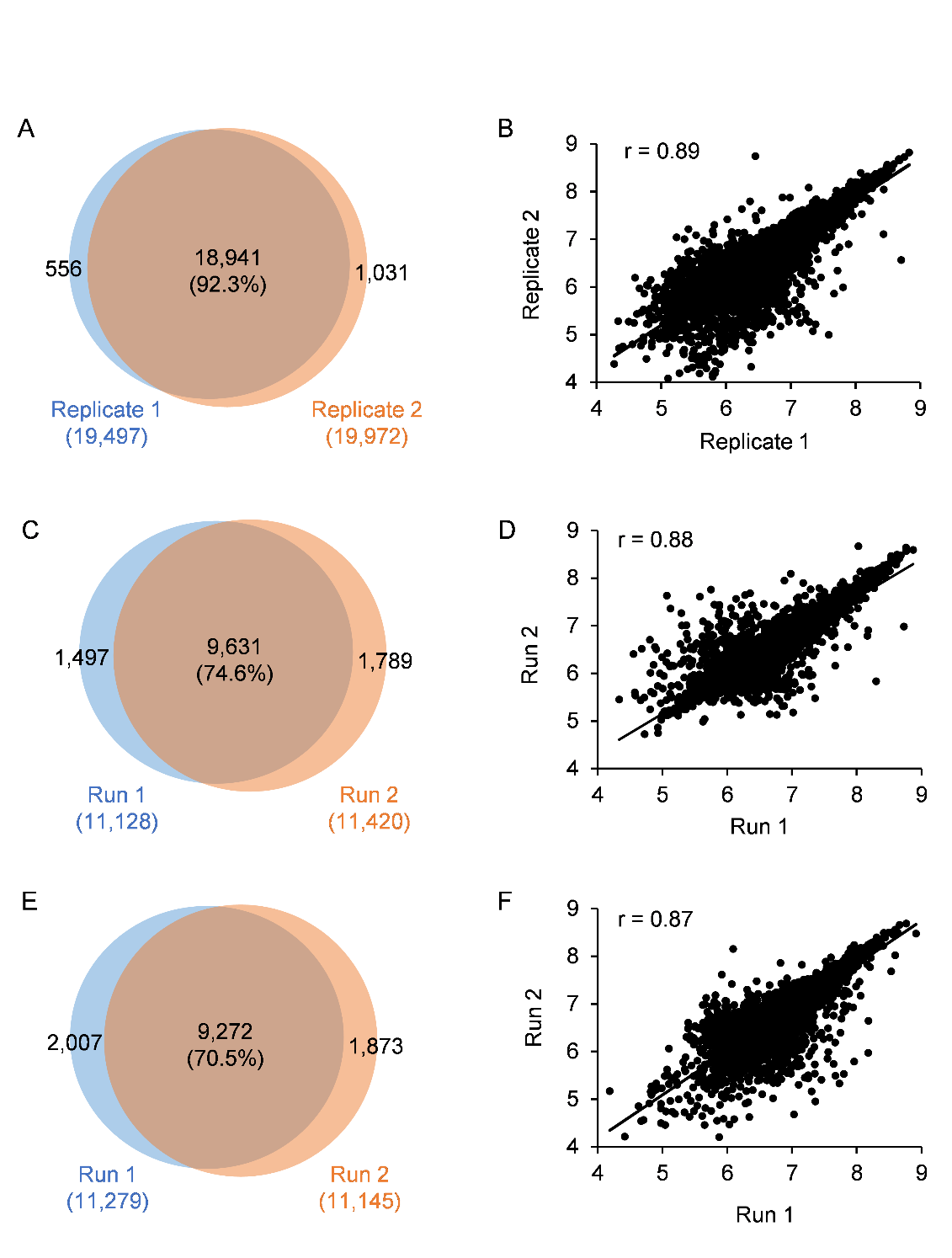


Supplementary Figure S2


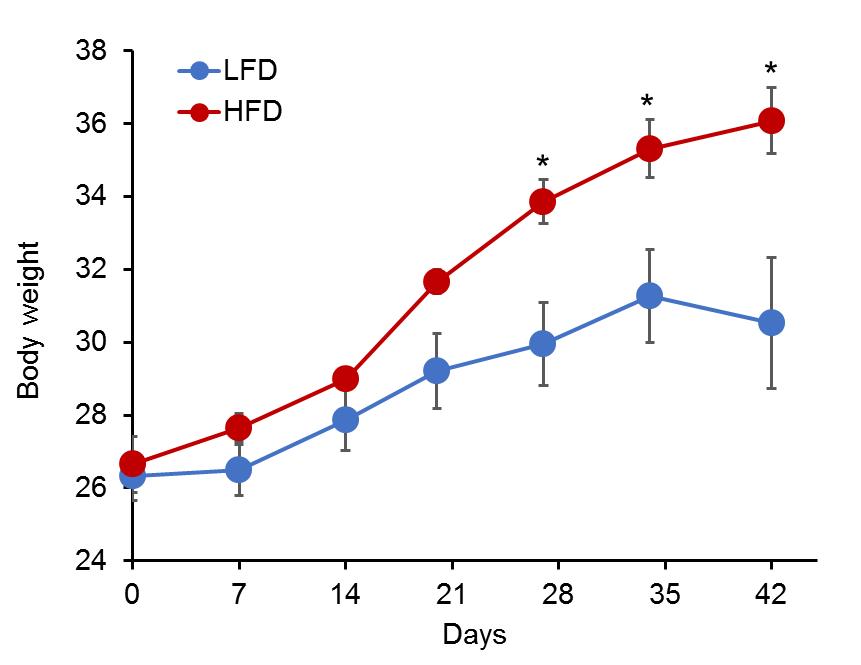


Supplementary Figure S3


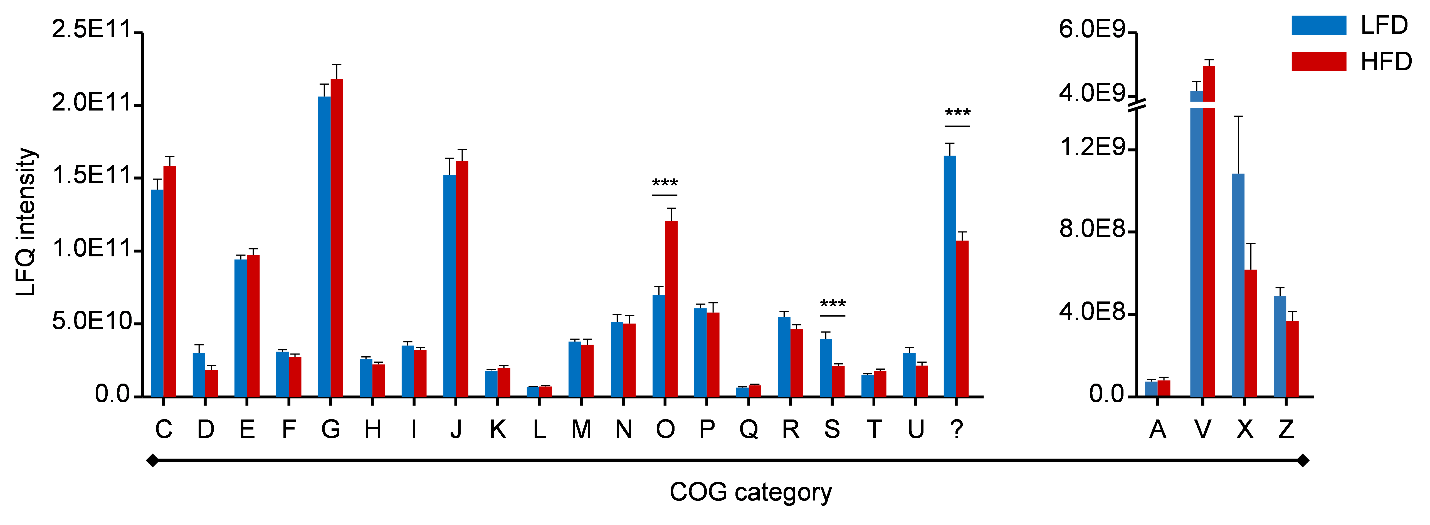


Supplementary Figure S4


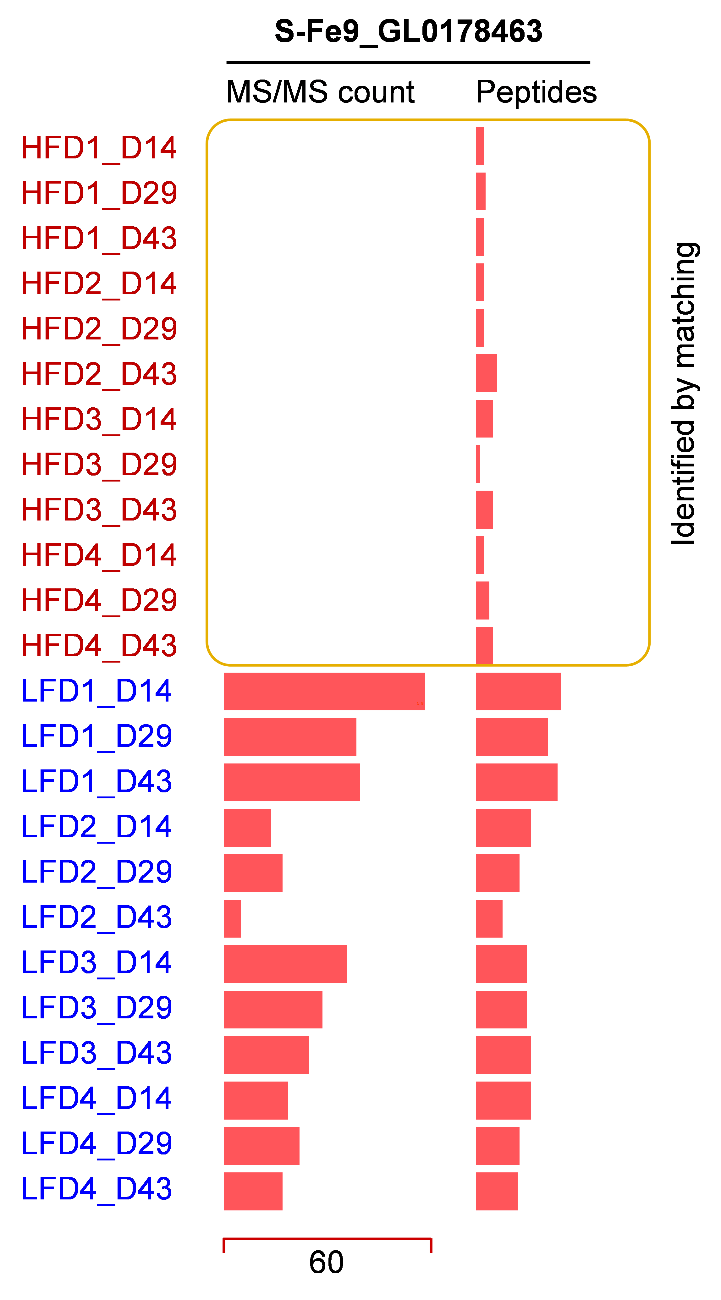

Supplement: Additional file 2: Figure S1–S4. — Figure S1. Analysis of murine fecal metaproteome data in the study of Tanca et al. using MetaPro-IQ approach. (A) Venn diagrams depicting the overlap of identified peptides between the two replicates; (B) scatter plot illustrating the Pearson’s correlation between the LFQ intensity of all peptides identified in both replicates; (C) and (E) showing the overlap of identified peptides between two runs for replicate 1 and replicate 2, respectively; (D) and (F) showing the Pearson’s correlation between the LFQ intensity of all peptides identified in both runs for replicate 1 and replicate 2, respectively. Detailed information on the sample information of murine fecal metaproteome (MFM) data was described in the study of Tanca et al. Figure S2. Effects of diet on the mouse body weight gain. Statistical differences were examined with two-sample t test. Mean ± SD was shown. *p < 0.05. Figure S3. COG category distributions of mouse stool microbial proteins. The COG category distributions of all proteins were shown. LFQ intensity was used for the analysis, and mean ± SEM was plotted. Each letter shows one COG category according to the standard naming in NCBI website and also shown in Additional file 1: Table S7. Question mark (?) denotes proteins without a COG assignment. Statistical analysis was performed using a two-sample t test with a Benjamini-Hochberg FDR correction. ***FDR-corrected P < 0.001. Figure S4. MS/MS count and peptide distribution of protein S-Fe9_GL0178463. The number of identified MS/MS and peptides for each sample was shown in the bar chart. The yellow box highlighted the samples with peptide identification only by matching instead of by MS/MS. (DOCX 583 kb) [file 40168_2016_176_MOESM2_ESM.docx]
